# Comment on the article *Structure and mechanism of copper–carbonic anhydrase II: a nitrite reductase*

**DOI:** 10.1107/S2052252520016644

**Published:** 2021-01-18

**Authors:** Dimitrios Tsikas

**Affiliations:** a Institute of Toxicology, Core Unit Proteomics, Hannover Medical School, Carl-Neuberg-Str. 1, Hannover, 30625, Germany

**Keywords:** carbonic anhydrase, nitrite reductase, nitrous anhydrase, protein structure, enzyme mechanisms, inorganic chemistry

## Abstract

The paper discusses a recent paper [Andring *et al.* (2020). *IUCrJ*, **7**, 287–293] on the nitrite reductase and nitrous anhydrase activity of carbonic anhydrase.

Carbonic anhydrase (CA) is one of the oldest, most efficient and best investigated ubiquitous Zn^2+^-containing enzymes. CA catalyzes a very simple but vital reaction, *i.e.* the hydration of carbon dioxide, in mammals, plants and bacteria (Meldrum & Roughton, 1933 ▸). Rather surprisingly, over recent decades many additional physiological and pathological roles of CA have been discovered. A newly discovered CA activity is the bioactivation of inorganic nitrite (O=N—O^−^) to nitric oxide (NO), a signaling multiple-functional gaseous molecule in living organisms. Central to scientific research on CA has been its catalytic site that preferentially binds Zn^2+^, which is redox-inactive, and Cu^2+^, which is redox-active (Lindskog & Nyman, 1964 ▸; Coleman, 1965 ▸). This topic is still of great scientific interest (Kim *et al.*, 2020 ▸). In addition, and in contrast to Zn^2+^, Cu^2+^ binds to two different centers of CA which are differently affected by gluta­thione (GSH), the most abundant endogenous intra-cellular antioxidant with high specificity to Zn^2+^, Cu^2+^ and other divalent ions including Hg^2+^ (Tabbì *et al.*, 2019 ▸). It can be expected that Cu^2+^-carrying CA is likely to exert not only the classical carbonic anhydrase activity, but may also be involved in redox-dependent reactions and mechanisms. For example, Cu^2+^-containing CA could oxidize NO to nitrite and higher nitro­gen oxides (NO_*x*_), as performed by the Cu^2+^-rich ceruloplasmin, or it could reduce nitrite to NO via intermediate Cu^+^-formation by GSH or ascorbic acid (Tabbì *et al.*, 2019 ▸). Such a reaction is practically impossible for regular Zn^2+^-containing CA.

Recently, Andring and associates reported the crystal structure of copper (II)-bound human carbonic anhydrase II (Cu^2+^-hCAII) in complex with inorganic nitrite (O=N—O^−^) at 1.2 Å resolution with two Cu^2+^ centers, analogous to bacterial nitrite reductases, and suggested that Cu^2+^-hCAII can function as a nitrite reductase, yet without providing experimental evidence (Andring *et al.*, 2020 ▸). In the *scientific commentary* on this article, Liljas stated that ‘Andring *et al.* (2020 ▸) have been able to unravel the mystery’ (Liljas, 2020 ▸), probably referring to the controversy that Aamand *et al.* (2009 ▸) found Zn^2+^-CAII to reduce nitrite to NO, whereas Andring *et al.* (2018 ▸) failed to detect Zn^2+^-CAII-mediated reduction of nitrite to NO.

Our studies using bovine and human Zn^2+^-CAII demonstrated formation of *S*-nitroso-gluta­thione (GSNO) from nitrite and GSH suggesting nitrous anhydrase activity of Zn^2+^-CAII, which was not inhibitable by the CA-inhibitors acetazolamide or dorzolamide (Hanff *et al.*, 2016 ▸; Zinke *et al.*, 2016 ▸). We observed formation of NO only in the presence of l-cysteine (CysSH), most likely due to the intermediate formation of *S*-nitro­socysteine (CysSNO), which can readily and abundantly decompose to NO in the presence of Cu^+^ (Tsikas *et al.*, 2002 ▸).

Cu^2+^ ions were found to bind to Zn^2+^-CAII isolated from human erythrocytes at a site other than the active site and inhibited the exchange of water from the enzyme without affecting the equilibrium rate of hydration of CO_2_ (Tu *et al.*, 1981 ▸). This observation may suggest that classical CA inhibitors such as acetazolamide may inhibit the carbonic anhydrase activity of CA by tightly binding to the CAII-bound Zn^2+^, through the sulfone amide group, but not to the second Cu^2+^-binding site. This could be an explanation for our observation that neither acetazolamide nor dorzolamide inhibited the nitrous anhydrase activity of bovine and human CAII (Hanff *et al.*, 2016 ▸; Zinke *et al.*, 2016 ▸).

Andring *et al.* (2020 ▸) stated that ‘recent reports have shown that CAII can also reduce nitrite (NO_2_^−^) to nitric oxide (NO)... (Andring *et al.*, 2018 ▸; Aamand *et al.*, 2009 ▸; Hanff *et al.*, 2018 ▸)’, that ‘However, when dialyzed with ethyl­enedi­amine­tetra­acetic acid (EDTA), the enzyme retained its carbonic anhydrase activity yet lost its nitrite reductase activity (Hanff *et al.*, 2018 ▸)’, and that ‘Furthermore, if this bovine CAII was dialyzed against EDTA, the nitrite reductase activity was ablated indicating that a metal cofactor within the bovine blood was needed for the CAII-dependent nitrite reductase activity (Andring *et al.*, 2018 ▸; Hanff *et al.*, 2018 ▸).’. We wish to point out this mistake in the paper by Andring *et al.* (2020 ▸). In the paper referred to above (*i.e.*, Hanff *et al.*, 2018 ▸), we did not report that CAII is a nitrite reductase, but we explicitly stated that we measured nitrous anhydrase activity of bovine and human CAII and CAIV, and did not use EDTA (*i.e.* Hanff *et al.*, 2018 ▸).
